# A clinical student exchange program organized by cardiothoracic department: feedback of participants

**DOI:** 10.1186/1749-8090-8-56

**Published:** 2013-03-29

**Authors:** Theodor Tirilomis, Friedrich A Schoendube

**Affiliations:** 1 Department for Thoracic, Cardiac, and Vascular Surgery, University of Goettingen, Robert-Koch-Str. 40, 37075, Goettingen, Germany

**Keywords:** Medical education, Medical student, Communication, International cooperation, Cardiovascular

## Abstract

**Background:**

The development of a student exchange program was an essential part of the cooperation between the Medical Schools of the University of Goettingen (Germany) and the University of Thrace in Alexandroupolis (Greece). The student exchange program started in 2008 and was performed once a year. The experiences of this program and the feedback of participants are presented.

**Methods:**

Although organized by the Dept. of Thoracic, Cardiac, and Vascular Surgery, the approach of the program was multidisciplinary. Participants also attended Continuous Medical Education activities primary addressed to physicians. At the end of the program, the participants evaluated the program anonymously. The educational units were rated via a 4-grade system. Additionally, it was possible to comment both positive and negative aspects of the program.

**Results:**

Twenty-nine educational units were evaluated. The practical teaching units yielded a better result than the classical teaching units (93% of practical units were evaluated as “very good” vs. 74% of lectures/seminars). The Continuous Medical Education activities were evaluated less favorable (only 61% were evaluated as “very good”).

**Conclusions:**

The student exchange program enhanced effective teaching and learning. Courses supporting practical medical skills were extremely positive evaluated. Continuous Medical Education activities are not suitable for students and therefore, we do not include such an event anymore. Additionally, the program created an excellent forum for contact and communication between the students of the two universities.

## Background

Globalization and internationalization are two modern keywords, but they increasingly gain importance in many areas of social life. In this context, increased mobility and flexibility make globalized education mandatory and desirable. Therefore, it is extremely important to implement collaborations in medical education. Existing cooperation is based mostly on individual level but it is essential to establish cooperation at “higher” levels too; at university level the cooperation may be between institutes, departments, schools/faculties, or even between entire institutions. Beginning with cooperation on department level, in 2008 an enlarged cooperation between the Faculty of Medicine of the Georg-August University Goettingen (Germany) and the Medical School of the Democritus University of Thrace in Alexandroupolis (Greece) started. An important part of this cooperation was the development and the implementation of a student exchange program. Therefore, a group of 10 students from the German University were invited to Alexandroupolis for a week and consequently 10 students of the Greek University were invited to Goettingen two months later. The students from Goettingen visited Alexandroupolis in October and the students from Alexandroupolis came to Goettingen in December.

The aims of this project were (i) the creation of a bi-national meeting forum for medical students, (ii) medical teaching and training using established and new teaching methods, (iii) the promotion of practical skills, and (iv) the increase of interest in medical research. This report presents the organization and the evaluation results of the second part of this program realized in Goettingen.

## Methods

The student exchange program was performed in 3 consecutive years. Thirty-one medical students of the University in Alexandroupolis (10 men and 21 women) participated in the exchange to Goettingen. The students were in the 3^rd^, 4^th^, and 5^th^ academic year. Conveniently, these students where hosted by the students from Goettingen, who, in turn, visited Greece themselves in October. The students were, furthermore, involved in the planning of the program. In addition to the teaching program, enough time was considered for social encounters and exchange.

**Figure 1:**
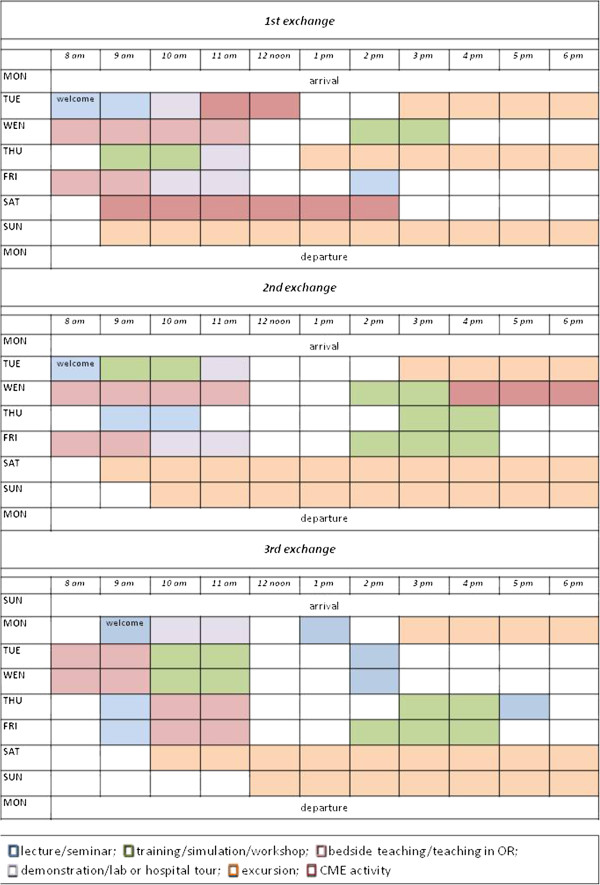
**Schedule of the academic events of the 1**^**st**^**, 2**^**nd**^**, and 3**^**rd **^**student exchange program ([light blue square] lecture/seminar; [light green square] training/simulation/workshop; [light brown square] bedside teaching/teaching in OR; [light gray square] demonstration/lab or hospital tour; [light orange square] excursion; [brown square] CME activity).**

The duration of the student exchange program was for the first and second program 8 days (from Monday to Monday) but for the third program a day more was added resulting in Sunday to Monday (Figure 1).

The educational program in Goettingen consisted of (i) lectures, (ii) seminars, (iii) bedside teaching (practical clinical), (iv) simulator teaching, (v) teaching in the operating room (OR), and (vi) workshops on basic surgical techniques. The lessons were taught mostly in English, occasionally in Greek or German. There have been a total of 29 teaching units (Table 1). The contents of the teaching units were drawn from the fields of cardiac surgery, thoracic surgery, vascular surgery, general surgery, cardiology, gastroenterology, anesthesiology, emergency medicine, intensive care medicine, pediatric cardiology, neonatology, and medical education. Additionally, continuing medical education (CME) events taking place at that time were also included in the program. These CME events were primarily addressed to physicians, being conducted mainly in German. Three CME activities were offered; the workshop “Minimally invasive mitral valve surgery”, consisting of live-surgery from the OR, the workshop “Vascular surgery”, consisting of anastomosis training sessions, and the symposium “Thoracic surgery”.

**Table 1:** Summary of the teaching units

	**n=**	**Teaching hours**
Lecture	7	7
Seminar	2	2
Simulator teaching	7	9
Bedside teaching	3	12
Teaching in operating room	3	15
Medical equipment teaching	1	1
Workshop	3	12
Presentation of research projects and laboratories	3	3

At the end of the exchange program the educational units and the program itself were evaluated by the participants. One participant of the third program did not return the evaluation form. The evaluation was anonymous and the educational units were rated via a 4-grade system: (1) “very good”, (2) “good”, (3) “acceptable”, and (4) “poor”. In addition, there was the possibility to comment both positive and negative features/aspects (Table 2).

**Table 2:** Examples of questions of the evaluation sheet

***Items and grades***				
- the lecture *“… –title- …”* was	- **o***very good*	- **o***good*	- **o***acceptable*	- **o***poor*
- the workshop *“… –title- …”* was	- **o***very good*	- **o***good*	- **o***acceptable*	- **o***poor*
- teaching in the OR was	- **o***very good*	- **o***good*	- **o***acceptable*	- **o***poor*
- I found positive:				
- I found negative:				
- Comments:				

OR: operating room.

The study was conducted in accordance with the regulations of the University Ethics Committee.

## Results

**Figure 2:**
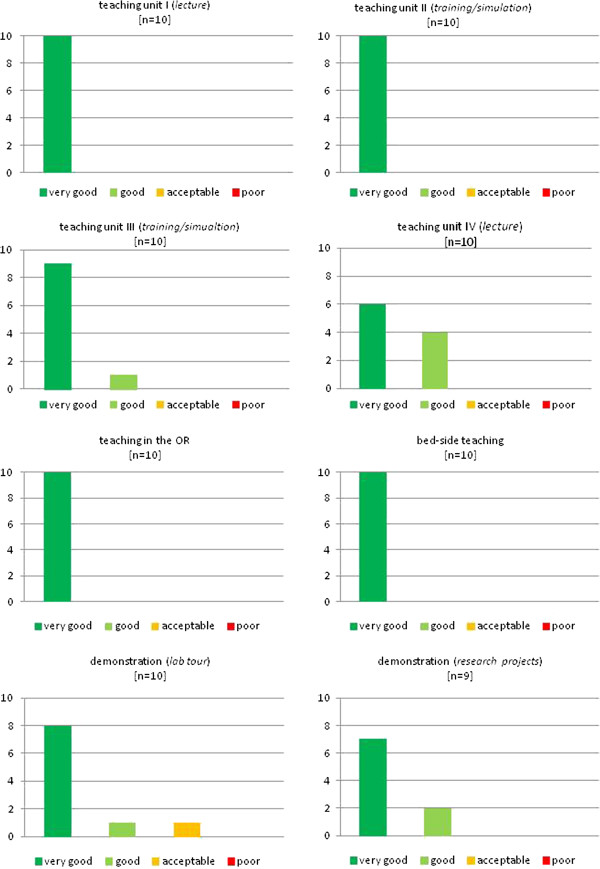
**The evaluated teaching units of the 1**^**st **^**student exchange program.**

**Figure 3:**
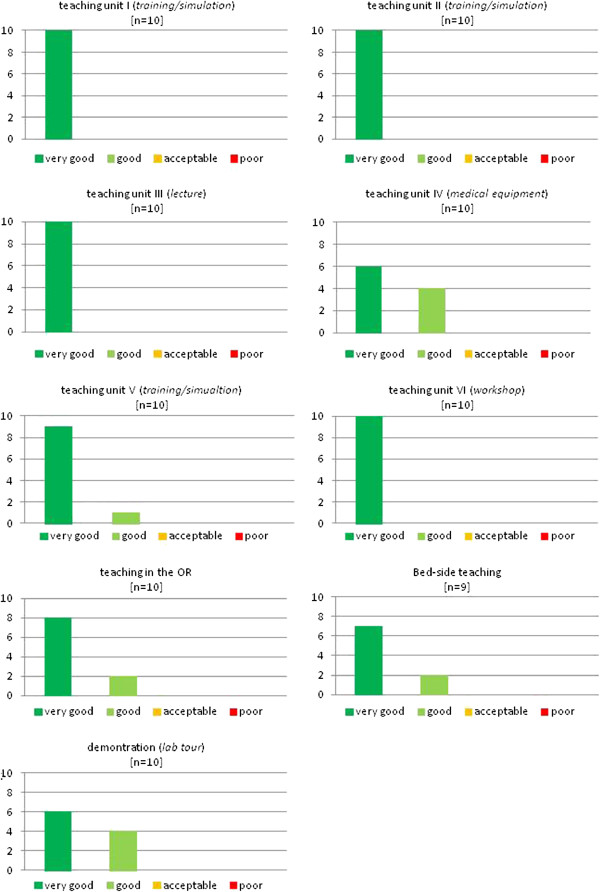
**The evaluated teaching units of the 2**^**nd **^**student exchange program.**

**Figure 4:**
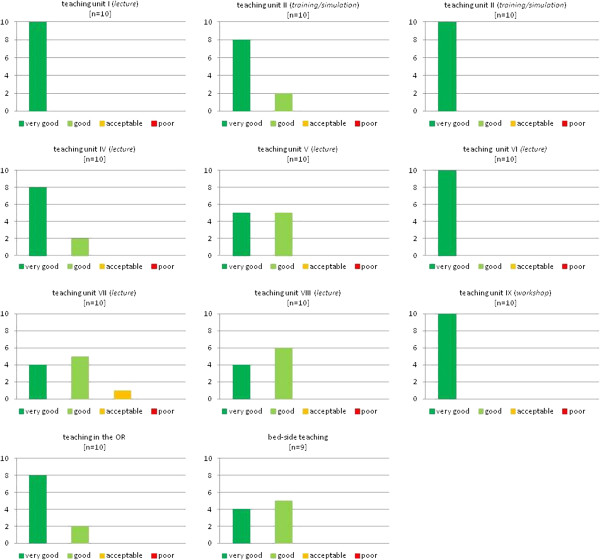
**The evaluated teaching units of the 3**^**rd **^**student exchange program.**

**Figure 5:**
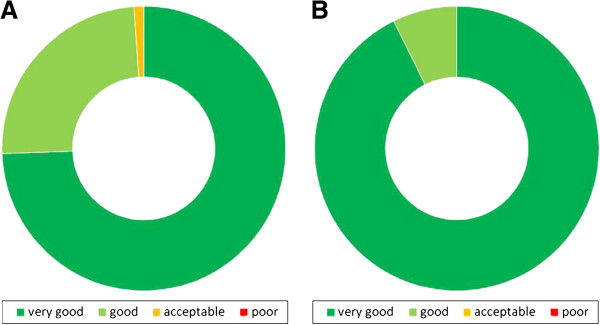
**Scoring results of different types of teaching units.** (**A**) **Scores for lectures and seminars (n = 90).** (**B**) **Scores of practical courses, including workshops for students (n = 109).** CME activities are not included.

The evaluation scores for each teaching unit are presented in Figures 2, 3, and 4. The evaluation shows better results for the practical units compared to the classical teaching forms of lectures and seminars. While only 74% of students graded the latter with “very good”, the former reached significantly higher results, scoring 94% in this category (Figure 5).

**Figure 6:**
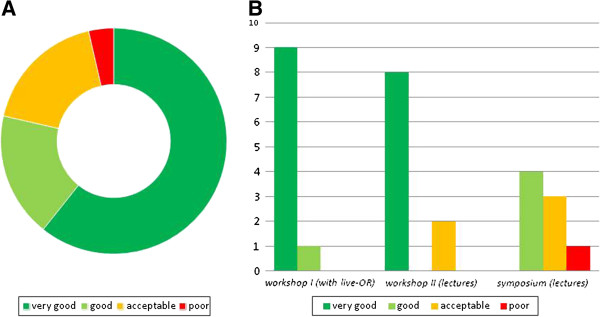
**Scores of medical students for CME activities primary addressed to physicians (n = 28); (A) Global score of CME units.** (**B**) **The scores of each individual unit; the “workshop I with live-OR” was held in English, the lectures of “workshop II” and “symposium” were in German.**

The CME activities were rated less favorably by the students (61% “very good”; Figure 6), mostly due to the language of the activity (5 negative comments); while the first workshop was held in English due to invited speakers from abroad, the other two events were conducted in German.

## Discussion

While international cooperation in research is common nowadays, cooperation in clinical teaching is rather exceptional. The most popular kind of international “cooperation” in the field of clinical education of medical students are the clinical elective (“Famulatur”) and parts of the internship (“Practical Year”). Such programs are based on the individual commitment of the students and their individual organizational ability. Furthermore, several other factors are important to the realization of this cooperation: the attractiveness of the host country and its non-medical environment, the reputation of the host country’s medicine, the program organization, and last but not least the costs [1]. Student organizations are increasingly active in organizing such activities [2]. Although a fairly good network of interested students (mostly organized by the respective student association), the contents of the educational activity organized abroad for elective periods are still very individual.

Beyond the educational activity frame of internship and clerkship, European students may participate in an Erasmus program [3]. Sometimes, collaborations may establish within the framework of various research projects and occasionally between multiple institutions; sometimes even across the continents [4]. In such projects individual students are incorporated mainly in the current academic program of the host university. However, this requires at least a sufficient command of the language of the host country. This linguistic problem can be avoided with the creation of specialized “summer or winter schools”. The language of these “schools” is mostly English. Courses offered in such a framework are often open to students from different countries and the participants deal intensively with a specific theme, e.g. cancer [5]. However, cooperation at higher academic level can be achieved by collaboration of departments or even university faculties. In the context of cooperation between the Faculty of Medicine in Goettingen and the Medical School in Alexandroupolis, a student exchange program was an essential part of the cooperation project. The program was open to all medical students of the clinical part (beyond the second academic year) and was announced in the Medical School. Additionally, the announcement was send by e-mail to the students. The students had had the opportunity to pre-register for participation via regular mail and e-mail. If more than 10 interested students were registered before the deadline, a lottery decided participation. After the deadline, if places remained free, the policy was “first come first serve”.

Educational contents were based on the Learning Curriculum of the Faculty of Medicine (Goettingen Catalog of Learning Objectives for the Clinical Part of Medical Study) [6]. The objectives were adapted for the group because it consisted of students having completed different amounts of clinical years and we did not want over- or under-demand the participants. The evaluation results show that this goal was achieved. Especially the courses supporting practical medical skills were extremely positive evaluated. For these courses it was important that all practical issues were held in English. This clearly indicates the importance not only of the contents of courses but also of the language of teaching.

Moreover, the primary goal of the program, the establishment of a forum for the students of two universities, has proven a success. According to the comments of the participants communication was not only limited to learning and educational purpose. A few weeks after the student exchange program a Greek participant wrote in his blog: “… Another surprise for us was undoubtedly the daily live of our German fellow students; we can say that there is no student who does not practice at least one extracurricular activity. Many of them are active members of various voluntary, non-profit organizations. ....” [7], this comment exemplifies the successful achievement of the goals of our academic education according to our academic curriculum: “… we support the development of our medical students into competent, self-learning, and responsible individuals” [6].

The student exchange program was also challenging for the academic teachers. New ideas, new methods, and new perspectives enrich both, learning and teaching. The medical teachers applied and modified classical and modern teaching methods, stimulating learning, knowledge, and discussion. New knowledge can be incorporated either directly in the patient’s treatment, or indirectly through new innovative research projects.

In general, the experience of the project showed that the organization and the coordination of such a program are very demanding and time-consuming. We could realize the program only through the individual commitment of the persons involved. For the success of such a program it is essential to give to the organizing persons enough space within the daily clinical work. For the future, we will ask the central academic institutions of the Faculty for technical support to improve the quality of coordination of the program. Nevertheless, we will keep the organization of the program in our hands to maintain a high degree of flexibility. Another important topic is the financing of such a project; oftentimes, this constitutes a major problem. Our program was primarily funded by the Department of Thoracic, Cardiac, and Vascular Surgery with the support from within the Faculty; the first time by the Department for International Relations of the Medical Faculty and the second time by the Dean’s Office. Finally, it was also important to give the program a formal frame; therefore, the participants of the program were welcomed to the University of Goettingen on the first day by both, the Dean (or Vice-Dean), and the director of the organizing Department. Moreover, in the evening, the mayor welcomed the students to Gottingen; this reception took place in the historic Town Hall.

An important lesson learned was that issues of CME activities are not suitable for students, presumably because the topics were too specialized. Therefore, we do not include such an event anymore.

## Conclusion

Through the organization and implementation of an exchange program for medical students, we reached an excellent transfer of knowledge and created a meeting forum for open communication. The next goal of the academic cooperation will be the creation of common research projects.

## Competing interests

The authors declare that they have no competing interests.

## Authors’ contribution

TT conceived of the study, participated in the design of the study, performed the data analysis, and drafted the manuscript. FAS participated in the design of the study and helped to draft the manuscript. Both authors read and approved the final manuscript.
